# Comprehensive N-Glycan Profiling of Cetuximab Biosimilar Candidate by NP-HPLC and MALDI-MS

**DOI:** 10.1371/journal.pone.0170013

**Published:** 2017-01-10

**Authors:** Sheng Liu, Wenjie Gao, Yao Wang, Zhenyu He, Xiaojun Feng, Bi-Feng Liu, Xin Liu

**Affiliations:** 1 Britton Chance Center for Biomedical Photonics at Wuhan National Laboratory for Optoelectronics-Hubei Bioinformatics & Molecular Imaging Key Laboratory, Systems Biology Theme, Department of Biomedical Engineering, College of Life Science and Technology, Huazhong University of Science and Technology, Wuhan, China; 2 Wuhan Centers for Disease Prevention and Control, Wuhan, China; Universidade de Sao Paulo, BRAZIL

## Abstract

Monitoring glycosylation of the mAbs have been emphasized and routinely characterized in biopharmaceutical industries because the carbohydrate components are closely related to the safety, efficacy, and consistency of the antibodies. In this study, the comprehensive glycan profiling of a biosimilar candidate of cetuximab was successfully characterized using Normal phase high-performance liquid chromatography (NP-HPLC) in combination with Matrix assisted laser desorption/ionization mass spectrometry (MALDI-MS). The presence of minor N-linked glycans containing sialic acid lactone residues (NeuAcLac) was observed in the biosimilar for the first time, which could influence the quantitative analysis of sialylated glycans and interfere with quantification of neutral glycans when it was analyzed by high performance liquid chromatography fluorescence (HPLC-FL). To overcome this issue, mild alkali treatment was used to hydrolyze lactone of the sialic acid to their neutral formation, which had no impact on the analysis of other glycans before and after the treatment. As a result, the mild alkali treatment might be helpful to obtain quantitative glycan profiling of the mAbs drugs with enhanced accuracy and robustness.

## 1. Introduction

**Fig 1:**
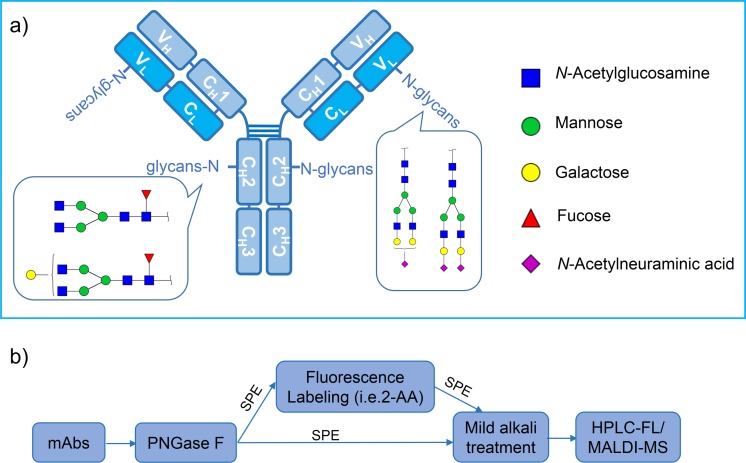
a) A representative schematic structure of monoclonal antibody and *N*-glycosylation sites on it. The main glycan moieties of the Fab and Fc fragment were shown in the frame. Structures and the monosaccharides are depicted following the CFG notation; b) flowchart of our method in this study.

Therapeutic recombinant monoclonal antibody (mAbs) drugs have emerged as a clinically important drug class, and more than 30 therapeutic antibodies have been approved for clinical use [1]. However, development of biosimilars is becoming a trend due to the coming off-patent of approximate 50% alternative of the existing mAbs and the expensiveness of the production and characterization of mAbs. All currently approved mAbs are based on IgGs and are most usually produced with the use of mammalian expression systems, such as mouse myeloma NS0, Chinese hamster ovary (CHO), and mouse myeloma Sp 2/0 cell lines [2–4]. Typical mAbs are comprised of two identical light chains and two identical heavy chains subunits interconnected by intramolecular disulfide bonds (Fig 1A). A conserved N-glycosylation site was contained in the C_H_2 domain at Asn297 and about 30% of polyclonal human IgG molecules bear N-linked oligosaccharides in the Fab region [5–7].

It has been well documented that the attached N-glycans on mAbs play crucial roles in many biological and physicochemical processes such as enhancement of the structural integrity, resistance against protease, effectiveness of serum half-life in vivo, and antibody-dependent cellular cytotoxicity [8–12]. For example, the sialic acids, a family of 9-carbon monosaccharides found as terminal residues on many glycan structures attached to glycoproteins, were closely related to some biological process such as cell–cell adhesion, cell surface receptor recognition, and progression of human malignancies [13–15]. In addition, non-human N-glycans could be presented in the non-human expression systems which will be a considerable risk in relation to immunogenicity and possible factor for incidence of some disease, and possible masking of existing antigenic sites on the peptide backbone causes a crucial side effect or insufficient efficacy [16]. Therefore, N-glycan profiling analysis of the mAbs is growing in importance and becoming a critical process in antibody characterization and regulatory evaluation [17].

High-performance liquid chromatography (HPLC) has been a conventional method for routine monitoring of N-glycosylation during process development and quality control of mAbs [18]. However, it suffers from unstable identification of structurally diverse glycans and could not provide direct information on glycan structure. Modern biological mass spectrometry (bio-MS) techniques based on soft ionization methods, such as matrix-assisted laser desorption/ionization mass spectrometry (MALDI-MS) has provided a powerful platform for the qualitative analysis of glycans from mAbs, thanks to its unique features of enhanced sensitivity, high throughput and high-molecular-weight detection abilities [19]. However, inaccurate glycan quantification could occur because the ionization efficiency was different for neutral and acid glycans [20]. Thus, several orthogonal techniques must be used to identify and quantify glycoforms, glycosylation profiling and carbohydrate contents of the mAbs.

In this study, we determined the glycan profiling of a biosimilar of cetuximab, which was produced in CHO cell lines in our lab, in a detailed structure and quantitative manner using NP-HPLC and in combination with MALDI-MS. However, several abnormal *N*-linked glycans containing NeuAcLac residues were observed in the biosimilar by MALDI-MS which was not reported previously. NanoLC-ESI-MS/MS was employed to elucidate the detailed structural information of minor aberrant glycans. It should be noted that even the amount of the unusual glycans were limited, their existence directly impacted on the accuracy of the quality control. In addition, the aberrant glycans were relatively stable after an overnight digestion in slight alkali buffer. Therefore, ammonium hydroxide solution (pH 10.0) was employed to transform the NeuAcLac containing glycans to their natural forms, which could be removed by rotary concentrator (as shown the flowchart of our method in Fig 1B). More importantly, it could not influence the quantitative analysis of total glycan pattern. With this simple treatment, a more accurate and robust quantification of glycans was obtained for the biosimilar. From the results obtained in this study, we strongly believe that the mild alkali treatment might be a prerequisite step for not only the accurate quantitative analysis but also the qualitative analysis of the glycans in therapeutic antibodies.

## 2. Materials and Methods

### 2.1. Chemicals and reagents

The biosimilar of cetuximab (IgG1 type) used in this paper was expressed in transfected CHO cells lines by our lab. The biosimilar of bevacizumab was given by Wuhan Xinshengye Co. Limited (Wuhan, China), which was also expressed by CHO cell lines. Peptide-N-glycosidase F (PNGase F) and endoglycosidase buffer kit were obtained from LCP Biomed (Lianyungang, China). Dimethyl sulfoxide (DMSO), 2-aminobenzoic acid (2-AA), 2-Picoline-borane (2-PB), trifluoroacetic acid (TFA), porous graphitic carbon (PGC) cartridges, microcrystalline cellulose (MCC) and ammonium hydroxide were purchased from Sigma-Aldrich (St. Louis, MO, U.S.A). Glacial acetic acid, ethanol, 1-butanol were obtained from Aladdin (Shanghai, China). LC-MS grade Formic acid (FA), pure water for NanoLC ESI-MS and Pierce^TM^ Fab Preparation Kit were attained from Thermo Fisher Scientific (Waltham, MA, U.S.A). Cetuximab, LC-MS grade Acetonitrile (ACN) and methanol were purchased from Merck KGaA (Darmstadt, Germany). All reagents were HPLC grade and all the reaction solutions were prepared with water purified by the Direct-Q system (Millipore, Bedford, MA).

### 2.2. Fragmentation of the cetuximab biosimilar

Fab and Fc fragments of the mAb biosimilar were prepared with Pierce^TM^ Fab Preparation Kit (Thermo Scientific) according to the manufacturer’s instruction. Briefly, 200 μL immobilized papain resin was equilibrated with 200 μL digestion buffer through centrifugation with spin columns. The equilibration step was repeated for three times. An amount of 200 μg (10mg/mL) mAbs diluted in 200 μL digestion buffer was added to the equilibrated immobilized papain and the sample was incubated for 8h with an end-over-end mixer at 37°C. After incubation, the digestion buffer with Fab and Fc fragments was collected by centrifugation at 2000 *g* and the resin was washed with 100 μL PBS for two times for optimal recovery. The Fab and Fc fragments was then applied to an equilibrated NAb Protein A Plus Spin Column and incubated with end-over-end mixing for 10 min. The flow through fraction containing Fab fragments was collected with a new 1.5 mL collection tube by centrifugation at 2000 *g* and wash column with 100 μL PBS for two more times. The Fc fragments was collected by washing the Protein A Plus Spin Column with IgG elution buffer and also repeated for two more times.

### 2.3. N-glycan release and purification

N-glycans of the intact cetuximab, biosimilar, Fab and Fc fragments of the biosimilar were enzymatically cleaved with N-glycosidase F according to previously published procedure with little modification [21]. 100 μg of the mAb and cetuximab were dissolved in 90 μL of sodium phosphate (50 mM, pH 7.5, LCP Biomed, China) containing 0.2% SDS and 10 mM dithiothreitol. The sample was incubated at 100°C for 10 min prior to adding 10 μL of 10% Nonidet P-40. The reaction mixture was incubated with PNGase F (10 units) for 18 h at 37°C. Following digestion, sample was then boiled for 5min to deactivate the enzyme. The released glycans were purified using PGC cartridges as previously reported [22]. Briefly, the sample was diluted with 0.5 mL water and subsequently purified using PGC cartridge. The cartridge was initially washed with 3 mL of ACN and 3 mL of 80% (v/v) ACN containing 0.1% (v/v) TFA, followed by 3 mL of water. The sample was then loaded on the PGC cartridge and washed with 3 mL of water to remove impurity and salts. Finally, sample was eluted with 1.0 mL of 40% (v/v) ACN containing 0.1% (v/v) TFA. The eluent was collected and the fraction was dried by a rotary concentrator (Hamburg, Germany) for further analysis. The dried glycans from intact mAb were also treated with 50 μL mild ammonium hydroxide (pH 10) at room temperature for 1h, which was then dried by rotary concentrator for further analysis.

### 2.4. Fluorescence labeling and purification of 2-AA derivatized oligosaccharides

2-AA labeling of glycans from intact biosimilar, Fab and Fc and mild ammonium hydroxide treated were conducted as previously reported with minor modifications [23]. Briefly, the dried glycans were mixed with 25 μL freshly prepared labeling solution (4.8 mg/mL 2-AA in DMSO containing 30% glacial acetic acid) and 25 μL freshly prepared reducing agent (10.7 mg 2-picoline-borane in DMSO). The mixture was shaked for 30 s and incubated at 65°C for 2 h. After incubation, the glycan derivatives were diluted with 0.5 mL of equilibration solution (1-butanol/H_2_O/ethanol (4:1:1, v/v/v)) and then purified using a self-packed MCC SPE [24]. Briefly, the self-packed MCC cartridges were first washed with 3.0 mL of water to prevent contamination by cellulose-derived materials into the eluent and then equilibrated with 3.0 mL binding solution of 1-butanol/H_2_O/ethanol (4:1:1, v/v/v). The reaction mixture was diluted in 500 μL binding solution and then applied to the cartridge. The cartridge was washed with 1 mL binding solution for three times to remove the excessive derivative reagents and other impurities. Finally, the Asn-glycan derivatives were eluted with 1 mL of ethanol/H_2_O (1:1, v/v) and dried by a rotary concentrator.

### 2.5. NP-HPLC analysis of 2-AA labeled oligosaccharides

The 2-AA labeled glycans were reconstituted in 40 μL of solution consisting of ACN/50 mM HCOONH4 (1:4, v/v) for NP-HPLC analysis. Samples were separated by TSK-Gel Amide-80 5 μm 4.6×250 mm column (Tosoh, Bioscience Shanghai Co, LTD) on a Shimadzu LC-20 AD separation module (Shimadzu, Milford, MA) equipped with a Shimadzu temperature control module and a Shimadzu RF-10AXL fluorescence detector. The column temperature was set at 40°C. Solvent A was 50 mM ammonium formate adjusted to pH 4.4 with formic acid solution and solvent B was acetonitrile. A 65-min run was used as keeping solvent B at 70% for 4 min at a flow rate of 1 mL/min and then the linear gradient of 70–50% solvent B over 59 min, followed by 1 min at 50–0% B and 3 min at 0% B, returning to 70% B over 1 min. Fluorescence detector was set with excitation and emission wavelengths of 360 and 419 nm, respectively [25]. Finally, all the glycan fractions were automatically collected based on the elution time of the peaks using an automatic fraction collector, dried in Speedvac concentrator and analyzed by nanoLC-MS/MS.

### 2.6. MALDI-TOF MS analysis

Matrix-assisted laser desorption ionization time-of flight mass spectrometry (MALDI-TOF-MS) analysis was performed using an Applied Biosystems 4800 Proteomics analyzer (AB SCIEX, Concord, Canada) that was equipped with a 355 nm Nd:YAG laser. The spectrometer was operated in the positive reflectron mode. DHB matrix was prepared in 50% ACN aqueous solution with a final concentration of 10 mg/mL. To suppress potassium adduct formation in the mass spectra, 10 mM sodium acetate aqueous in 1:1 methanol: water was applied to dissolve the dried sample. 0.5 μL samples mixed with 0.5 μL of freshly prepared DHB matrix were directly loaded onto the stainless steel MALDI plate and allowed to dry in a gentle stream of warm air. Samples were ablated with a power of 4000 while the laser rastered over the target surface. A total of 1,000 laser shots were employed in each sample spot. The MS data processing was further performed by DataExplorer 4.0 (AB SCIEX, Concord, Canada).

### 2.7. NanoLC-ESI-MS and MS/MS analysis

The 2-AA labeled glycans and native glycans were analyzed using Triple TOF 5600 mass spectrometer (AB SCIEX, USA) equipped with a NanoLC system (NanoLC Ultra, Eksigent, Dublin, CA). The 2-AA labeled glycans were enriched on-line using a trap column (150 μm i.d. × 10 mm long; C18, 5 μm; Eksigent, Dublin, CA) and then separated using a 25min gradient on the analytical column (75 μm i.d. × 100 mm long; 5 μm; Eksigent, Dublin, CA) at a flow rate of 300 nL/min. A gradient condition was employed, i.e., 95–70% A for 10 min, 70–40% A for 5 min, 40–20% for 5 min and 95% A for 10 min for the 2-AA labeled glycans. Native glycans were enriched on-line using a trap column (150 μm i.d. × 1cm long; PGC, 5 μm; Proteomics Front, China) and then analyzed by a separation column (75 μm i.d. × 10 cm long; PGC, 5 μm; Proteomics Front, China.). A 50 min gradient was used for the native glycan analysis: 0 min 5% solvent B; 5 min5% solvent B; 30 min 30% solvent B; 40 min 80% solvent B; 41 min 95% solvent B; 45 min 95% solvent B; 46 min 5% solvent B; 50 min 5% solvent B. The solvent A consisted of 5% ACN solution containing 0.1% FA (v/v), and the solvent B consisted of 95% ACN solution containing 0.1% FA (v/v). For the two columns, the MS was all manipulated in positive-ion mode with a nano ion spray voltage typically maintained at 2.3 kV, and the source temperature was set at 150°C. The MS scanning range was acquired from 500 to 2,000 (m/z) with up to 20 precursors selected for MS/MS from m/z 100–2,000. MS scans were performed for 0.25 s, and the following 20 MS/MS scans were performed for 0.1 s each, which resulted in the full cycle for 2.8 s. Data were processed with PeakView 1.2 software (AB SCIEX, USA), and the elucidation of the glycans were edited by GlycoWorkbench 2.1 software [26].

## 3. Results and discussion

### 3.1. Glycans profiling analysis by MALDI-TOF MS

**Fig 2:**
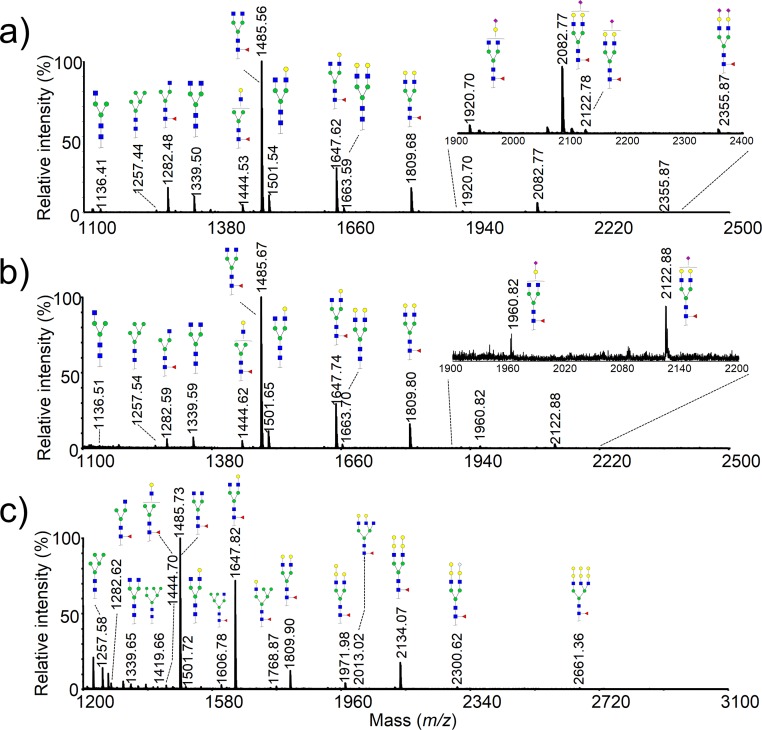
MALDI-TOF MS spectrum of N-glycans enzymatically released from the biosimilar of cetuximab and cetuximab. a) native N-glycans before mild alkali treatment (pH 10 ammonium hydroxide); b) native N-glycans of the biosimilar after mild alkali treatment; c) native N-glycans from the cetuximab. The cartoons of possible structures of glycans were adapted from Glycoworkbench and structure is depicted following the CFG notation.

Thanks to the advantages of ease of operation and accuracy in composition assignment of glycans, MALDI-MS is often used as a first step for glycan profiling analysis to generate information about the nature and diversity of glycans released from native, recombinant glycoproteins or even more complex biological samples. In this study, the native glycans released by PNGase F were enriched by PGC SPE prior to the direct MALDI analysis. The sample was handled by mass spectrometer in the positive reflectron mode. As shown in Fig 2A, the main glycans in the biosimilar of cetuximab were G0F, G1F, and G2F, which was in agreement with the glycan profile of cetuximab that shown in Fig 2C. However, there were still differences between the cetuximab and the biosimilar. Although two kinds of structures that could cause severe hypersensitivity reactions was not observed in the biosimilar, three sialylated glycans, proposed chemical composition of GlcNAc_4_Man_3_Gal_1_Fuc_1_NeuNAc_1_, GlcNAc_4_Man_3_Gal_2_Fuc_1_NeuNAc_1_ and GlcNAc_4_Man_3_Gal_2_ Fuc_1_NeuNAc_2_ with 18 or 36 Da loss were detected, which was corresponding to 1920.71, 2082.77 and 2355.87 with only one sodium ions adduct, respectively. The detailed structural information about the aberrant glycans was verified by nanoLC-ESI-MS/MS as mentioned below. Up to now, only a recent article described the appearance of dehydrated sialic acids in the serum of mouse [27]. We supposed that the sialic acid in the glycans were dehydrated and a structure of lactone (NeuAcLac) was formed. It is well known that lactone was unstable under alkali condition [28]. However, the NeuAcLac residues containing glycans were relatively stable in the biosimilar even after an overnight incubation (16–18 h) in the PNGase F digestion buffer (pH 7.5). Previously, we demonstrated that man-made lactone of sialic acids could be decomposed with pH 10 solution, which has minor influence to the glycan profiling [29]. Therefore, a slightly high pH ammonium hydroxide solution (pH 10) was used to accelerate hydrolysis of the lactone in the glycans, which could be later conveniently removed by evaporating in speed vacuum. The glycan profiling from the biosimilar after alkali treatment was shown in Fig 2B. However, due to the low ionization response and in or out postsource of sialylated glycans, only two sialylated glycans in free form was observed at *m/z* 1960.82 and 2122.88, corresponding to GlcNAc4Man_3_Gal_1_Fuc_1_NeuAc_1_ and GlcNAc_4_Man_3_Gal_2_ Fuc_1_NeuAc_1_ with two sodium ions adduct. Clearly, the above results verified our hypothesis. And the detailed glycan structures in the biosimilar were shown in S1 Table.

Other mAbs, a biosimilar of Bevacizumab, without dehydrated glycans was also used to evaluate the impact of mild alkali treatment to the glycan profiling. As shown in the S1 Fig, the chromatogram that obtained with and without mild alkali treatment are almost overlapped. In addition, the peaks from two of the chromatograms were collected and analyzed by nanoLC-ESI-MS, which showed the same glycan structures in the corresponding peaks (Data not shown). Furthermore, technical replicates were conducted (n = 3) to analyze the reproducibility of this method. Average CV for the N-glycans peaks was 2.39% before and after mild alkali treatment. These results indicated that the mild alkali treatment could not impact the glycan profiling with or without the esterified glycans.

### 3.2. Glycan profiling of 2-AA labeled from the biosimilar by NP-HPLC

HPLC with fluorescence detection is one of the most widely used detection method in the quality control of biopharmaceuticals due to the high sensitivity and possibility to estimate the relative amounts of glycans in glycoprotein. Since fluorescence detection does not allow for the direct structure elucidation, the identity of glycans must be confirmed either by MS or chromatography of labeled standards. However, the latter approach is limited by the number of commercially available standards. As the reasons presented above, NP-HPLC was applied in this study to quantitative analysis the 2-AA labeled glycans from the biosimilar and nanoLC combined with MS was used to determine the structure of glycans.

**Fig 3:**
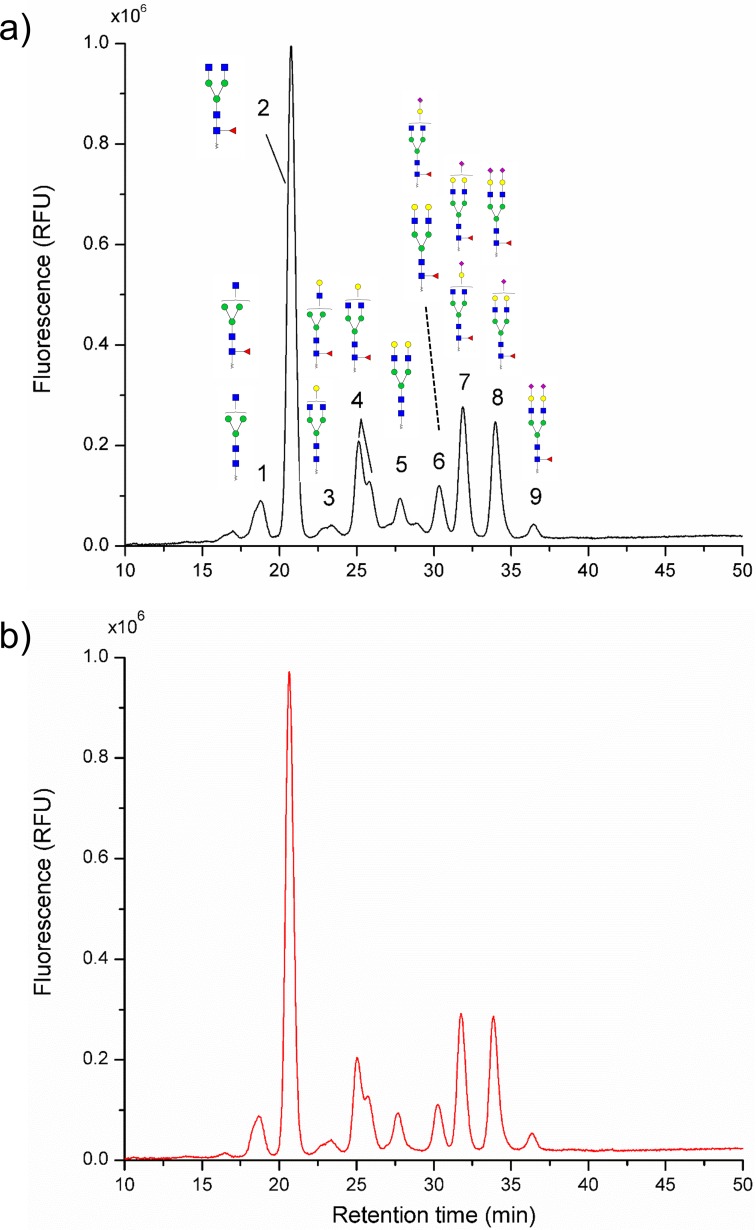
Typical NP-HPLC spectrum of 2-AA labeled glycans from the biosimilar of cetuximab. a) 2-AA labeled mAbs glycans before mild alkali treatment; b) 2-AA labeled mAbs glycans after mild alkali treatment.

The representative chromatogram of glycans from the biosimilar was shown in Fig 3A and the relative intensity of each peak was calculated and displayed in S2 Table. Four major peaks were observed in the chromatogram of the biosimilar, which represented the glycans GlcNAc_4_Man_3_Fuc_1_ (peak 2), GlcNAc_4_Man_3_Gal_1_Fuc_1_ (peak 4), GlcNAc_4_Man_3_Gal_2_Fuc_1_NeuAc_1_ (peak 7) and GlcNAc_4_Man_3_Gal_2_Fuc_1_NeuAc_2_ (peak 8), respectively. GlcNAc_4_Man_3_Gal_1_Fuc_1_ at peak 4 consists of two isomeric glycans with the same molecular weight but different oligosaccharide structural distribution, displayed in the chromatogram at different retention time. Relative peak area was utilized to quantitative analysis of the glycans in the biosimilar. Although there were no significant difference before and after mild alkali treatment in the chromatogram, the relative peak area of several peaks were still changed (Fig 3A and 3B). As shown in S2 Table, the percentage of peak 6 (including GlcNAc_4_Man_3_Gal_2_Fuc_1_ and GlcNAc_4_Man_3_Gal_1_Fuc_1_NeuAcLac_1_), changed from 7.12±0.70% to 5.66±0.23%, was strongly decreased. We also observed that the lactonization of the sialic acid in glycans could decrease the interaction with the solid phase of the column which shorten the retention time. There is no doubt that this could interfere with the quantitative analysis of the glycans. After the mild alkali treatment, the glycans with free sialic acid returned to their due position. Consequently, accurate quantitative analysis could be obtained using our simple mild alkali treatment method. In addition, each peak was collected by an automatic fraction collector and the structure information was further identified with nanoLC-ESI-MS/MS.

Although we initially used MALDI-TOF MS to confirm the molecular structure by mass assignment, we found that relative peak intensities detected in the glycan spectra were not well correlated with the relative abundance obtained by NP-HPLC (Figs 2A and 3A). It is general accepted that MALDI-MS does not allow real quantitative for oligosaccharides unless stable isotope-labeled analogs are incorporated as internal standards [30]. As a result, HPLC-FL might be the golden standard for the quantitative analysis of the glycans in the biopharmaceutical drugs.

### 3.3. NanoLC-ESI-MS and MS/MS analysis

**Fig 4:**
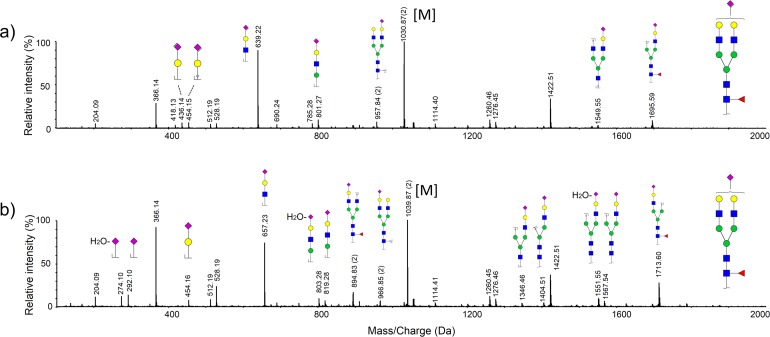
NanoLC-ESI-MS/MS spectrum of native glycans. a) MS/MS spectra of *m/z* 2060 with chemical composition of GlcNAc_4_Man_3_Gal_2_NeuAcLac_1_; b) MS/MS spectra of *m/z* 2078 with chemical composition of GlcNAc_4_Man_3_Gal_2_NeuAc_1_.

Mass-only compositional assignments could not obtain the detailed structural information of the glycans. In order to further elucidate minor glycans in the biosimilar, the structural information of the abnormal glycans were confirmed by the nanoLC-ESI-MS/MS. Although the CID-based tandem MS/MS often failed to produce sufficient number of cross-ring fragments, it could provide abundant B/Y ions. As shown in Fig 4, several sets of diagnostic ions between the normal and abnormal structures were shown in a typical MS/MS spectrometry. For example, *m/z* 436, 639, 801, 1549 and 1695, corresponded to fragment ions from the aberrant glycan moiety of GlcNAc_4_Man_3_Gal_2_NeuAcLac_1_, and *m/z* 454, 657, 819, 1567 and 1713, corresponded to fragment ions from the normal glycan moiety of GlcNAc_4_Man_3_Gal_2_NeuAc_1_. Clearly, the 18 Da mass shift of CID MS/MS fragment spectra demonstrated the different chemical component of aberrant and normal glycan. Spectrum was also screened for the presence of typical NeuAc-associated fragments in the normal glycans, including *m/z* 292 (NeuAc), 274 (NeuAc-H_2_O), and 657 (Hex_1_HexNAc_1_NeuAc_1_) [27]. In contrast, there was no typical NeuAc-associated diagnostic fragment in the abnormal N-glycans. These results indicated that minor glycans in the biosimilar were NeuAcLac-contained glycans. Structural assignment in the glycan structure and tandem MS/MS analysis was performed using the GlycoWorkbench suite [26].

### 3.4. Fab and Fc N-glycan analysis

**Fig 5:**
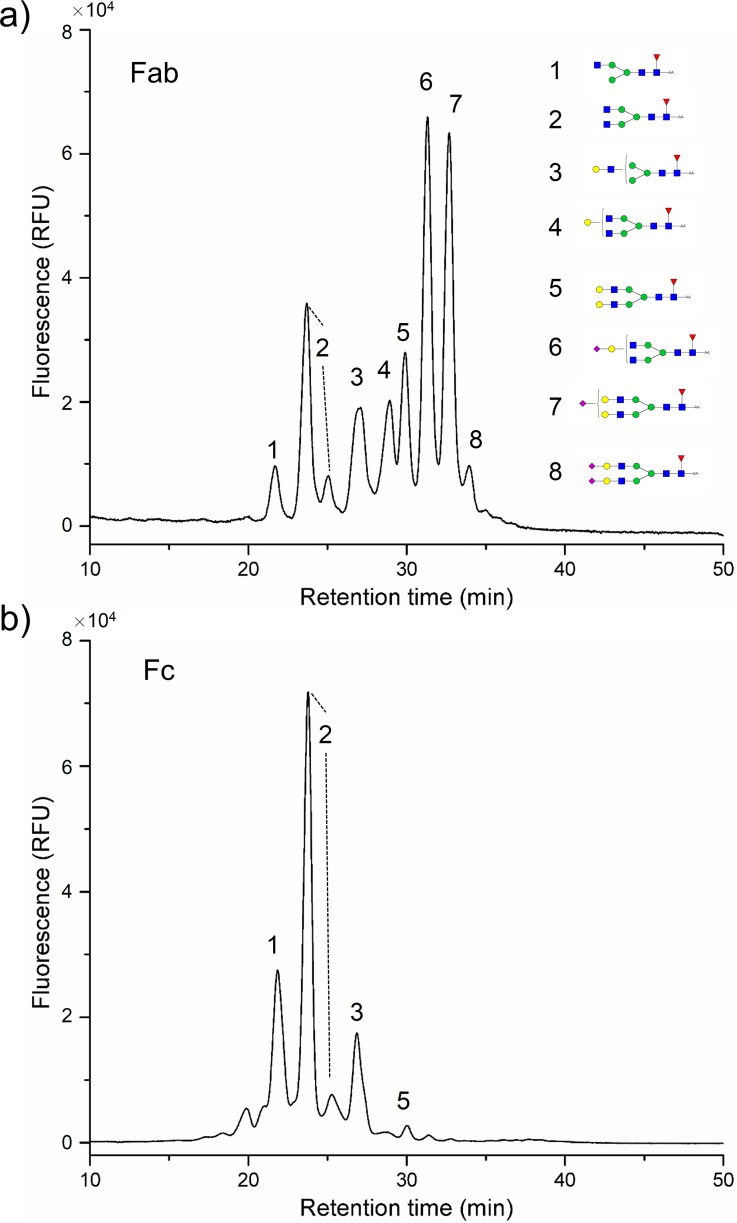
Typical NP-HPLC spectrum of 2-AA labeled glycans from the Fab and Fc fragment of the biosimilar of cetuximab. a) N-glycans on the Fab fragment; b) N-glycans on the Fc fragment. The compositions and structural schemes of glycans in each chromatographic peak are shown in S2 Table of the Electronic Supplementary Material.

Approximately 15–20% of IgGs bear N-linked oligosaccharides in the IgG Fab region, in addition to those attached at the conserved glycosylation site at Asn297 in the IgG Fc [8]. Our results demonstrated that the biosimilar of cetuximab also had two N-linked glycosylation sites. The Fab and Fc fragments of the mAb biosimilar were obtained by papain digestion and the glycans on the Fab and Fc fragments were released by PNGase F and labeled by 2-AA. The 2-AA labeled glycans in the Fab and Fc was then analyzed by NP-HPLC. The representative chromatogram of Fab and Fc fragment was shown in Fig 5. In addition, NanoLC-ESI-MS/MS was employed to identify 2-AA glycan structures in Fab and Fc fragments, which was shown in S3 Table. The major of the Fc glycans were core fucosylated complex biantennary oligosaccharides with zero, one, or two Gal residues and less than 5% of Fc glycans in the biosimilar are sialylated. From previously reported literature, Fc glycosylation was required for the induction of antibody-mediated effector functions including ADCC and CDC by altered the three-dimensional structure of the protein, and the terminal Gal content of the IgG affect the CDC [31]. The glycans in the Fab have been dominated by biantennary complex-type structures, in contrast to Fc glycans, which were highly sialylated. The Fab N-glycans could be involved in immunomodulation, because they influence the affinity and avidity of antibodies for antigens as well as antibody half-life [32, 33]. Since there were minor sialylated glycans on the Fc fragment, most of the sialylated glycans resulted from the Fab fragment bearing the NeuAcLac-contained glycans. The biological effects about the minor abnormal glycans from the biosimilar were not reported, and we focused on the accurate glycan profiling analysis of the biosimilar in this study. Potential risk of the NeuAcLac-contained glycans in the therapeutic antibody will be studied in the future.

## Conclusion

In this study, we identified the glycan profiling of one mAbs tested as biosimilar candidate of cetuximab which produced in CHO cell lines. NP-HPLC coupled to fluorescence detection in combined with MALDI-TOF MS analysis have allowed us to comprehensively identify and confirm the presence of glycans in the biosimilar. The major glycan moieties in the biosimilar were in agreement with the innovator of cetuximab. However, several minor aberrant N-linked glycans containing NeuAcLac residues were observed in the biosimilar which was not reported previously. In addition, NanoLC-ESI-MS/MS was employed to elucidate the altered minor glycans in the biosimilar. Glycans of the fragments (Fab and Fc) about the biosimilar were also investigated. Results showed that the sialylated glycans in the biosimilar were mainly in the Fab fragment. In order to get more accurate quantification of the biosimilar, a slightly alkali treatment using ammonium hydroxide was conducted to transform NeuAcLac residues containing glycans to their neutral forms. As the treatment could not impact the glycan profiling, we strongly suggested mild alkali treatment as a prerequisite step for accurate glycan quantification of the therapeutic recombinant.

## Supporting Information

S1 Fig HPLC spectrum of 2-AA labeled glycans from a mAb without lactone containing glycans before and after mild alkali treatment (a) and the relative percentage area of the peaks (b).
(DOC)

S1 Table Detected N-glycans from the biosimilar of cetuximab by MALDI-MS. (DOC)

S2 Table The major peaks of the biosimilar and their abundance (%) determined by NP-HPLC with 2-AA labeling before and after ammonium hydroxide treatment. (DOC)

S3 Table Major glycans detected in the Fab and Fc of the biosimilar. (DOC)
