# Supplementary material for: Comprehensive N-Glycan Profiling of Cetuximab Biosimilar Candidate by NP-HPLC and MALDI-MS
Source: PLoS One. 2017 Jan 10;12(1):e0170013. doi: 10.1371/journal.pone.0170013 (PMC5225015; doi:10.1371/journal.pone.0170013)
Supplement: S1 Fig — HPLC spectrum of 2-AA labeled glycans from a mAb without lactone containing glycans before and after mild alkali treatment (a) and the relative percentage area of the peaks (b). (DOC) [file pone.0170013.s001.doc]

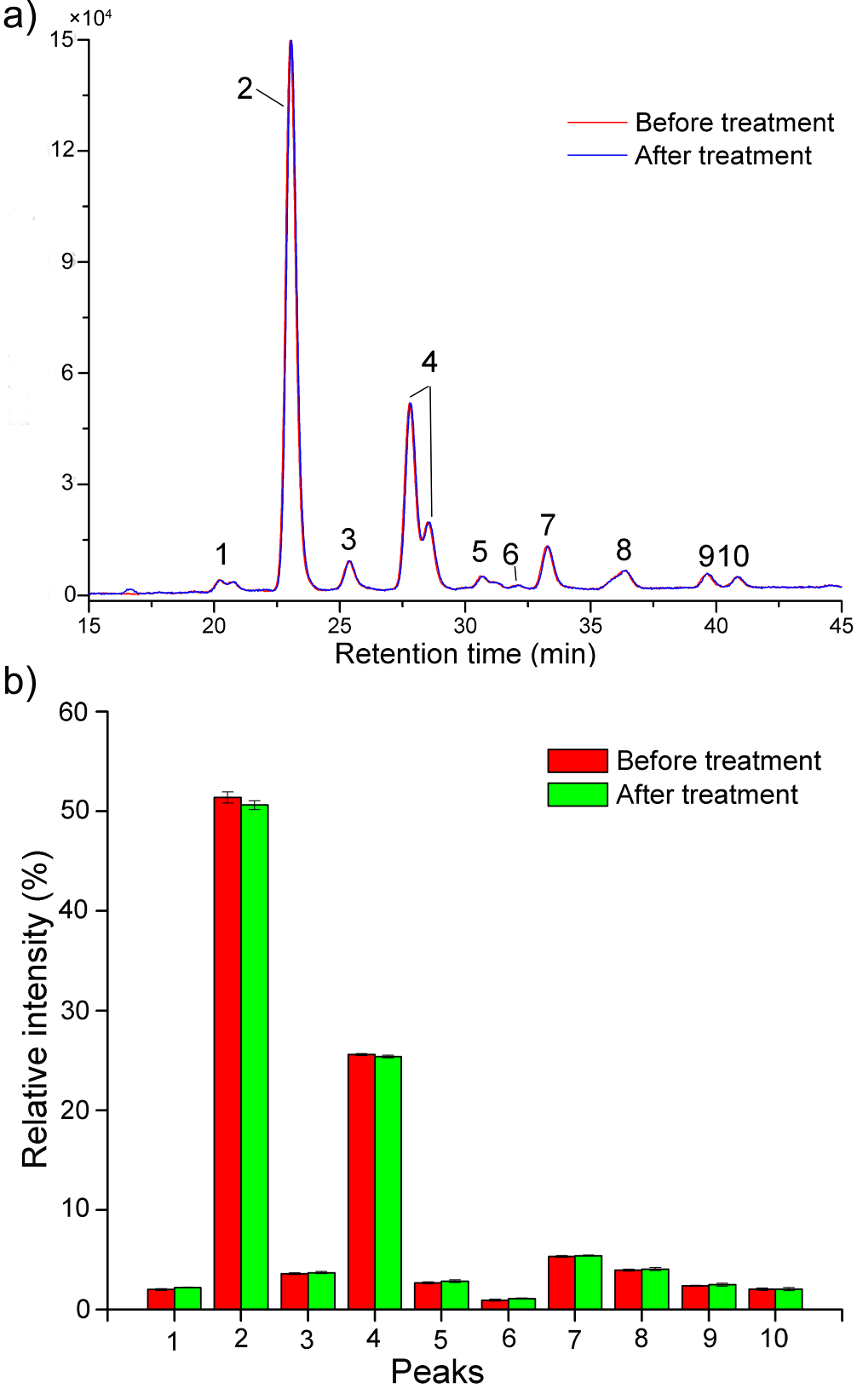


S1 Fig. HPLC spectrum of 2-AA labeled glycans from a mAb without lactone containing glycans before and after mild alkali treatment and the relative percentage area of the peaks.
