# Supplementary material for: Comprehensive N-Glycan Profiling of Cetuximab Biosimilar Candidate by NP-HPLC and MALDI-MS
Source: PLoS One. 2017 Jan 10;12(1):e0170013. doi: 10.1371/journal.pone.0170013 (PMC5225015; doi:10.1371/journal.pone.0170013)
Supplement: S1 Table — (DOC) [file pone.0170013.s002.doc]

**S1 Table**. Detected N-glycans from the biosimilar of cetuximab by MALDI-MS.

| **No.** | **Observed**  ***m/z*** | **Theoretical**  ***m/z*** | **Chemical**  **composition*** | **Structure**** |
| --- | --- | --- | --- | --- |
| 1 | 1136.41 | 1136.40 | H3N3 | 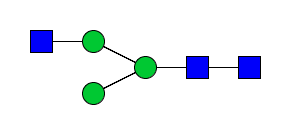 |
| 2 | 1257.44 | 1257.42 | H5N2 | 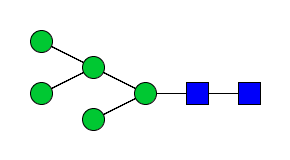 |
| 3 | 1282.48 | 1282.45 | H3N3F1 | 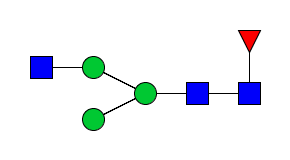 |
| 4 | 1339.50 | 1339.48 | H6N2 | 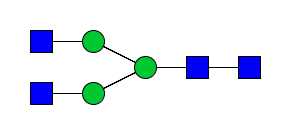 |
| 5 | 1444.53 | 1444.51 | H4N3F1 | 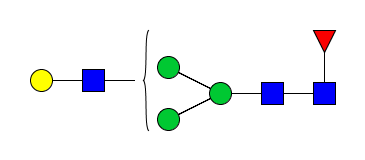 |
| 6 | 1485.56 | 1485.53 | H3N4F1 | 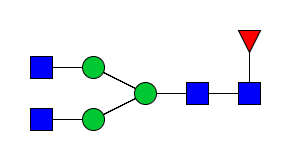 |
| 7 | 1501.54 | 1505.53 | H4N4 | 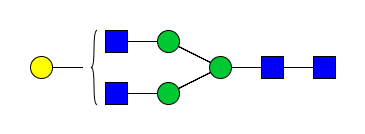 |
| 8 | 1647.82 | 1647.59 | H4N4F1 | 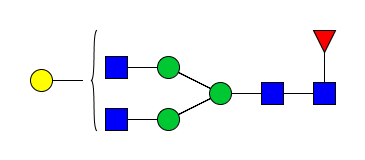 |
| 9 | 1663.59 | 1663.58 | H5N4 | 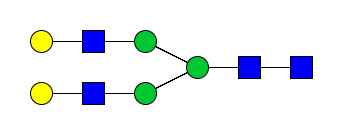 |
| 10 | 1809.68 | 1809.64 | H5N4F1 | 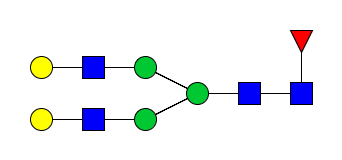 |
| 11 | 1920.71 | 1920.67 | H4N4F1L1 | 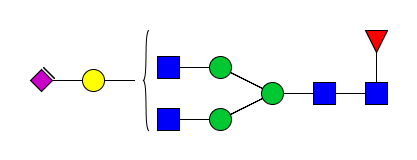 |
| 12 | 1960.82 | 1960.68 | H4N4F1S1 | 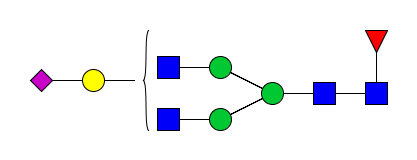 |
| 13 | 2082.77 | 2082.72 | H5N4F1L1 | 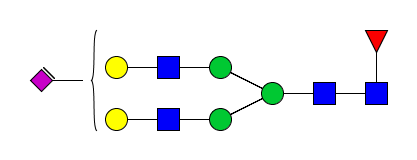 |
| 14 | 2122.88 | 2122.73 | H5N4F1S1 | 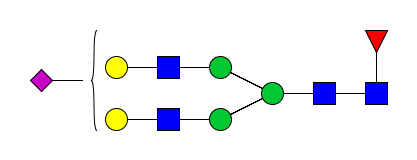 |
| 15 | 2355.87 | 2355.81 | H5N4F1L2 | 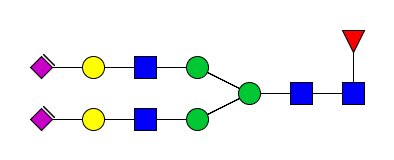 |

* Compositions are given as follows: hexose (H), N-acetylhexosamine (N), N-acetylneuraminic acid (S), fucose (F) and lactonization of α2,3-linkage N-acetylneuraminic acid (L) .

** Sugar residues are *N*-acetylglucosamine (
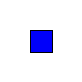
), mannose (
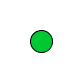
), galactose (
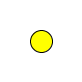
), Fucose (
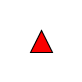
), *N*-acetylneuraminic acid(
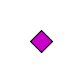
)　and *N*-acetyl neuraminic acid lactone (
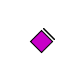
).
