# Supplementary material for: Comprehensive N-Glycan Profiling of Cetuximab Biosimilar Candidate by NP-HPLC and MALDI-MS
Source: PLoS One. 2017 Jan 10;12(1):e0170013. doi: 10.1371/journal.pone.0170013 (PMC5225015; doi:10.1371/journal.pone.0170013)
Supplement: S2 Table — (DOC) [file pone.0170013.s003.doc]

**S2 Table.** The major peaks of the biosimilar and their abundance (%) determined by NP-HPLC with 2-AA labeling before and after ammonium hydroxide treatment.

| Peak | Before ammonium hydroxide  treatment (*n*=5)* | After ammonium hydroxide  treatment (*n*=5)* |
| --- | --- | --- |
| 1 | 4.54±0.09 | 4.47±0.02 |
| 2 | 39.20±0.34 | 37.93±0.28 |
| 3 | 2.10±0.12 | 2.21±0.01 |
| 4 | 13.71±0.18 | 13.24±0.12 |
| 5 | 5.19±0.05 | 4.54±0.06 |
| 6 | 6.38±0.07 | 5.43±0.08 |
| 7 | 13.95±0.16 | 14.35±0.04 |
| 8 | 13.37±0.20 | 15.58±0.04 |
| 9 | 1.57±0.11 | 2.30±0.08 |

* Average ± standard deviations of *n* replicate runs.
