# Supplementary material for: Comprehensive N-Glycan Profiling of Cetuximab Biosimilar Candidate by NP-HPLC and MALDI-MS
Source: PLoS One. 2017 Jan 10;12(1):e0170013. doi: 10.1371/journal.pone.0170013 (PMC5225015; doi:10.1371/journal.pone.0170013)
Supplement: S3 Table — (DOC) [file pone.0170013.s004.doc]

**S3 Table**．Major glycans detected in the Fab and Fc of the biosimilar.

| **No** | **Observed**  ***m/z*** | **Theoretical**  ***m/z*** | **Chemical**  **Composition*** | **Structure**** |
| --- | --- | --- | --- | --- |
| Fab  1 | 1403.53 | 1403.51 | H3N3F1 | 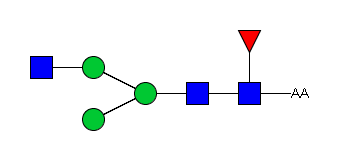 |
| 2 | 1606.60 | 1606.59 | H3N4F1 | 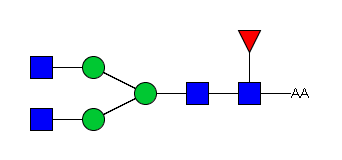 |
| 3 | 1565.58 | 1565.56 | H4N3F1 | 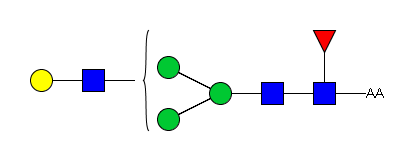 |
| 4 | 1768.64 | 1768.66 | H4N4F1 | 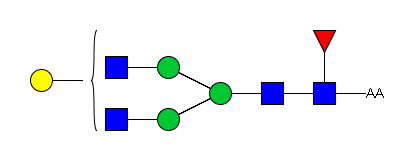 |
| 5 | 2059.75 | 2059.73 | H4N4S1F1 | 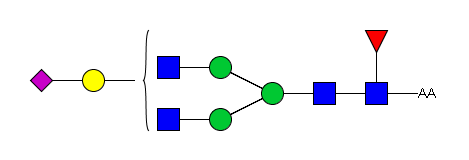 |
| 6 | 1930.71 | 1930.69 | H5N4F1 | 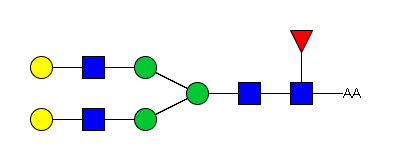 |
| 7 | 2221.81 | 2221.79 | H5N4S1F1 | 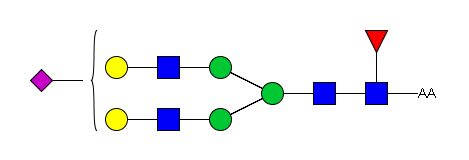 |
| Fc |  |  |  |  |
| 1 | 1403.53 | 1403.51 | H3N3F1 | 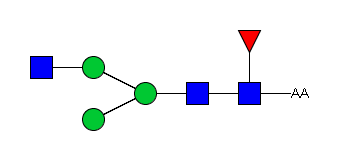 |
| 2 | 1606.60 | 1606.59 | H3N4F1 | 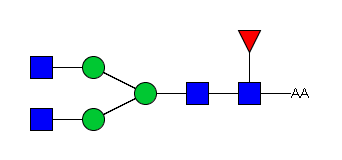 |
| 3 | 1565.58 | 1565.56 | H4N3F1 | 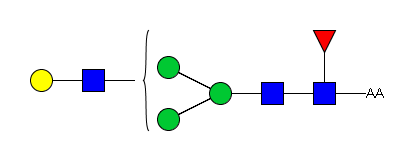 |
| 4 | 1930.71 | 1930.69 | H5N4F1 | 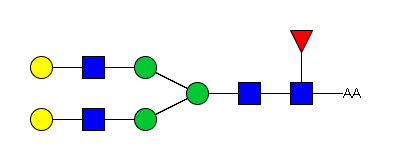 |

* Compositions are given as follows: hexose (H), N-acetylhexosamine (N), N-acetylneuraminic acid (S) and fucose (F).
